# Sevoflurane inhibits neuronal migration and axon growth in the developing mouse cerebral cortex

**DOI:** 10.18632/aging.103041

**Published:** 2020-04-09

**Authors:** Dongdong Chai, Jia Yan, Chunzhu Li, Yu Sun, Hong Jiang

**Affiliations:** 1 Department of Anesthesiology and Critical Care Medicine, Shanghai Ninth People’s Hospital Affiliated to Shanghai Jiao Tong University School of Medicine, Shanghai, China

**Keywords:** sevoflurane, neuronal migration, axon length, neuro-oncological ventral antigen 2 (Nova2), Netrin-1/Deleted in Colorectal Cancer (Dcc)

## Abstract

The highly organized laminar structure of the mammalian brain is dependent on successful neuronal migration, and migration deficits can cause lissencephaly and behavioral and cognitive defects. Here, we investigated the contribution of neuronal migration dysregulation to anesthesia-induced neurotoxicity in the fetal brain. Pregnant C57BL/6 mice at embryonic day 14.5 received 2.5% sevoflurane daily for two days. Cortical neuron migration and axon lengths were evaluated using GFP immunostaining. Morris water maze tests were performed to assess the effects of sevoflurane exposure on spatial memory in offspring. We found that sevoflurane exposure decreased axon length and caused cognitive defects in young mice. RNA sequencing revealed that these defects were associated with reduced neuro-oncological ventral antigen 2 (Nova2) expression. In utero electroporation experiments using Nova2 shRNA recapitulated this finding. Nova2 shRNA inhibited neuronal migration and decreased axon lengths. Finally, we found that Netrin-1/Deleted in Colorectal Cancer (Dcc) proteins acted downstream of Nova2 to suppresses neuronal migration. These findings describe a novel mechanism by which prenatal anesthesia exposure affects embryonic neural development and postnatal behavior.

## INTRODUCTION

To decrease the risk of spontaneous abortion and preterm labor, any surgery required by a pregnant woman is typically delayed until the second trimester when most of the physiological changes in the fetus have reached a plateau and anesthesia is relatively safer [1]. Protection of fetal development in utero is the prime concern for most parents when anesthesia is required for surgical procedures [2]. The second trimester is a critical period for development of the embryonic nervous system: neurogenesis, neuronal migration, and corticogenesis are the major neurodevelopmental events that occur at this stage [3]. Neuronal migration also plays an important role in nervous system formation and in neuronal development and activity [4]. Dysregulation of neuronal migration is associated with numerous neurological diseases, including cerebral malformation, epilepsy, autism, and schizophrenia [5]. Mounting evidence suggests that brain development is highly susceptible to surrounding environmental and drug effects during mid-gestation. In rodents, even a relatively nonintrusive operation such as abdominal ultrasonography can cause fetal neuronal migration deficits [6]. Maternal ethanol ingestion can also prevent neurons from migrating to the cortex and can impair cortical formation during embryonic development, leading to behavioral abnormalities [7]. In previous experiments, we have found that dual sevoflurane exposure can inhibit neuronal migration in offspring [8]; however, whether and how dual sevoflurane exposure affects spatial learning in those offspring remains unknown.

To date, a limited number of molecules that are known to be involved in neuronal migration in the developing cortex, such as N-cadherin, integrins, and connexins, have been identified [9]. Neuro-oncological ventral antigen 2 (Nova2), a neuronal-specific RNA binding protein, was first identified as an autoimmune antigens in the neurodegenerative disease POMA (paraneoplastic opsoclonus myoclonus ataxia). Nova2 proteins are widely expressed in several tissues, including in the nervous system [10], vascular endothelial cells [11], and pancreatic β cells [12]. Nova2 harbors three KH-type RNA binding domains, and binds directly to RNA sequences harboring YCAY motifs [13], which in turn regulate other processes including alternative splicing. Many potential Nova2 targets identified in genome-wide studies are involved in various neural developmental processes [14–16]. In vivo studies using Nova2 knockout mice have demonstrated defects in synapse formation function and neuronal migration [17–20]. Loss of Nova2 function reduces the migration of spinal cord interneurons progenitors and disturbs axon outgrowth and guidance in commissural interneurons [21]. In addition, recent studies demonstrated that Nova2^-/-^ knockout mice have axonal pathfinding defects, agenesis of the corpus callosum, and axonal outgrowth defects specific to ventral motor neuron axons and efferent innervation of the cochlea [13]. Given its role in neuronal migration and axon outgrowth, we postulated that Nova2 might also play a crucial role in neurotoxicity induced in offspring by dual sevoflurane exposure. In this study, we investigated the effects of dual sevoflurane exposure in mid-gestation on learning and memory in offspring and the underlying molecular mechanisms.

## RESULTS

### Dual sevoflurane exposure induced cognitive defects in young animals

Correct positioning of neurons during development achieved through directed migration is the basis for proper brain function. Previous research has demonstrated that neuronal migration is achieved through a rearrangement of cytoskeletal components in response to extracellular cues, which are mediated by numerous intracellular signaling pathways [22]. Neural development in mice is similar to that in humans, and 14 days of gestation in mice is equivalent to mid-gestation in humans. Although the neurodevelopmental timeframe differs between mice and humans, neural migration and axon outgrowth in the developing cerebral cortex are similar [23]. We therefore used 14-day-pregnant mice in our experiments.

**Figure 1:**
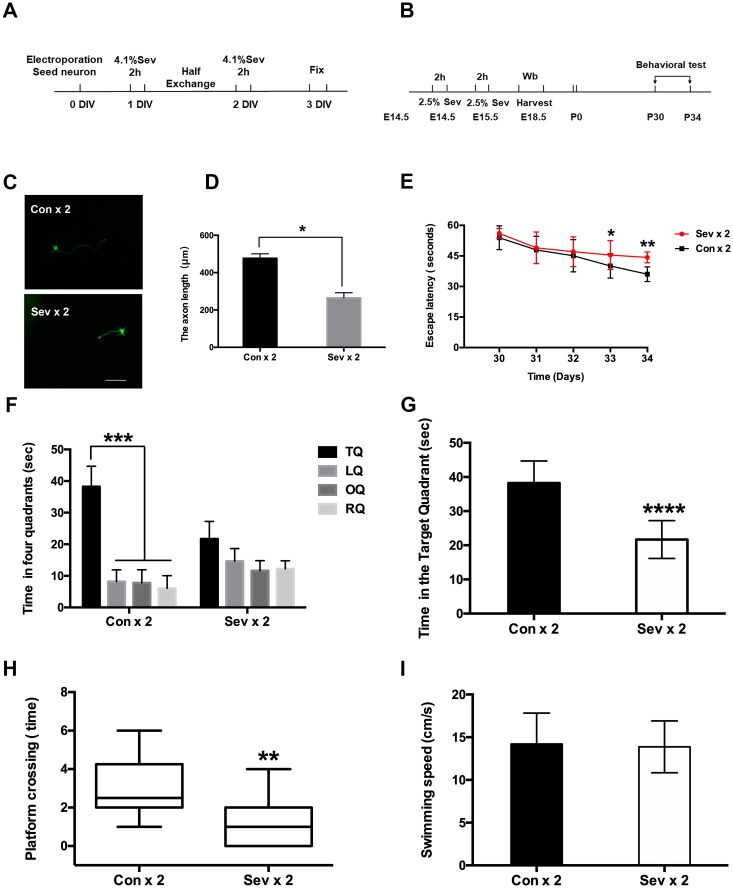
**Effects of sevoflurane anesthesia on spatial learning and memory in young mice.** (**A**) Flowchart of the neuron electroporation experiment. (**B**) Flowchart of the MWM experiment. (**C**) Dual sevoflurane exposure decreased axon length in primary cultured mouse cortical neurons. (**D**) The statistical results for the axon length between the two groups. Scale bars = 100 μm; approximately 70 cells from three independent experiments were counted during the statistical analysis (*P* = 0.0147*, Student’s t-test). (**E**) The escape latency on the 4^th^ day of acquisition training was increased in the sevoflurane group (Sev x 2 vs Con x 2, *F* = 0.828, *P* = 0.028*, Student’s t-test, N = 10). During the probe trial, the escape latency was also increased in the dual sevoflurane group (Sev x 2 vs Con x 2, *F* = 1.35, *P* = 0.007**, Student’s t-test, N = 10). (**F**) During the probe trial, the control group spent much more time in the target quadrant than other quadrants (*P* < 0.001***, N = 10, one-way ANOVA), while the sevoflurane group spent similar periods in the four quadrants (*P* > 0.05, N = 10, one-way ANOVA). TQ, LQ, OQ, and RQ is the target quadrant, the left quadrant, the opposite quadrant, and the right quadrant, respectively. (**G**) Dual sevoflurane exposure decreased the time spent in the target quadrant (*F* = 0.143, *P* < 0.0001****, N = 10, Student’s t-test). (**H**) Sevoflurane decreased the platform crossing times (*F* = 1.156, *P* = 0.0033**, N=10, Student’s t-test). (**I**) Sevoflurane did not affect swimming speed compared with the same variables in the control group mice. Data are expressed as the means ± S.D. *P < 0.05, **P<0.01, ***P<0.001.****P < 0.0001.

Previous experiments have concluded that dual sevoflurane exposure can affect the neuronal migration pattern in offspring [8]. Establishment of necessary connections in the nervous system relies on the ability of axons to locate and recognize their appropriate synaptic partners [24]. Leading axons must travel a sufficient distance to reach their final destination during neuron migration; axons length therefore plays an important role in neuronal function. Using a Nikon A1 fluorescence microscope equipped with a 20x objective to trace axonal length, we found that dual sevoflurane exposure in mid-gestation significantly decreased the axon length (Figure 1C, 1D, *P* = 0.0147, N = 3, Student’s t-test). Additionally, we found that six hours of clinical anesthesia administration during pregnancy didn’t affect neuronal migration levels or axon growth (Supplementary Figure 1B and 1D). Next, we investigated the effects of propofol and ketamine, two commonly used clinical intravenous anesthetics, on neuronal migration. Neither propofol nor ketamine had adverse effects on neuronal migration or axon growth after two separate two-hour infusion in fetal mice. (Supplementary Figure 1F and 1I).

Cognitive functions in young mice were assessed in the Morris Water Maze (MWM) test, which was used to measure spatial memory from P30 to P34. Escape latency is a major indicator of the capacity for spatial learning, while reference memory function is assessed in the probe trial [25]. Two-way ANOVA with repeated measurements showed a significant interaction between anesthesia exposure (sevoflurane versus control) and time (P30 to P34). Dual sevoflurane exposure induced cognitive impairment, as evidenced on P33, by increasing escape latency (Figure 1E, *P* = 0.028, N=10, Student’s t-test). During the probe trial (P34), escape latency was also increased in the dual sevoflurane exposure group (Figure 1E, *P* = 0.007, N=10, Student’s t-test). Mice in the control group also spent more time in the target quadrant during the probe trial (P34) (Figure 1F, *P* < 0.001, N = 10, one-way ANOVA), while sevoflurane group mice spent nearly equal amounts of time all four quadrants. Moreover, sevoflurane group mice spent significantly less time in the target quadrant than control group mice (Figure 1G, *P* < 0.0001, N = 10, Student’s t-test). In addition, platform crossing time also differed between the sevoflurane and control groups (Figure 1H, *P* = 0.0033, N=10, Student’s t-test). Swimming speed, however, was similar between the two groups (Figure 1I, *P* = 0.7721, N=10, Student’s t-test).

### Dual sevoflurane exposure-induced Nova2 deficiency contributes to neurotoxicity in offspring

**Figure 2:**
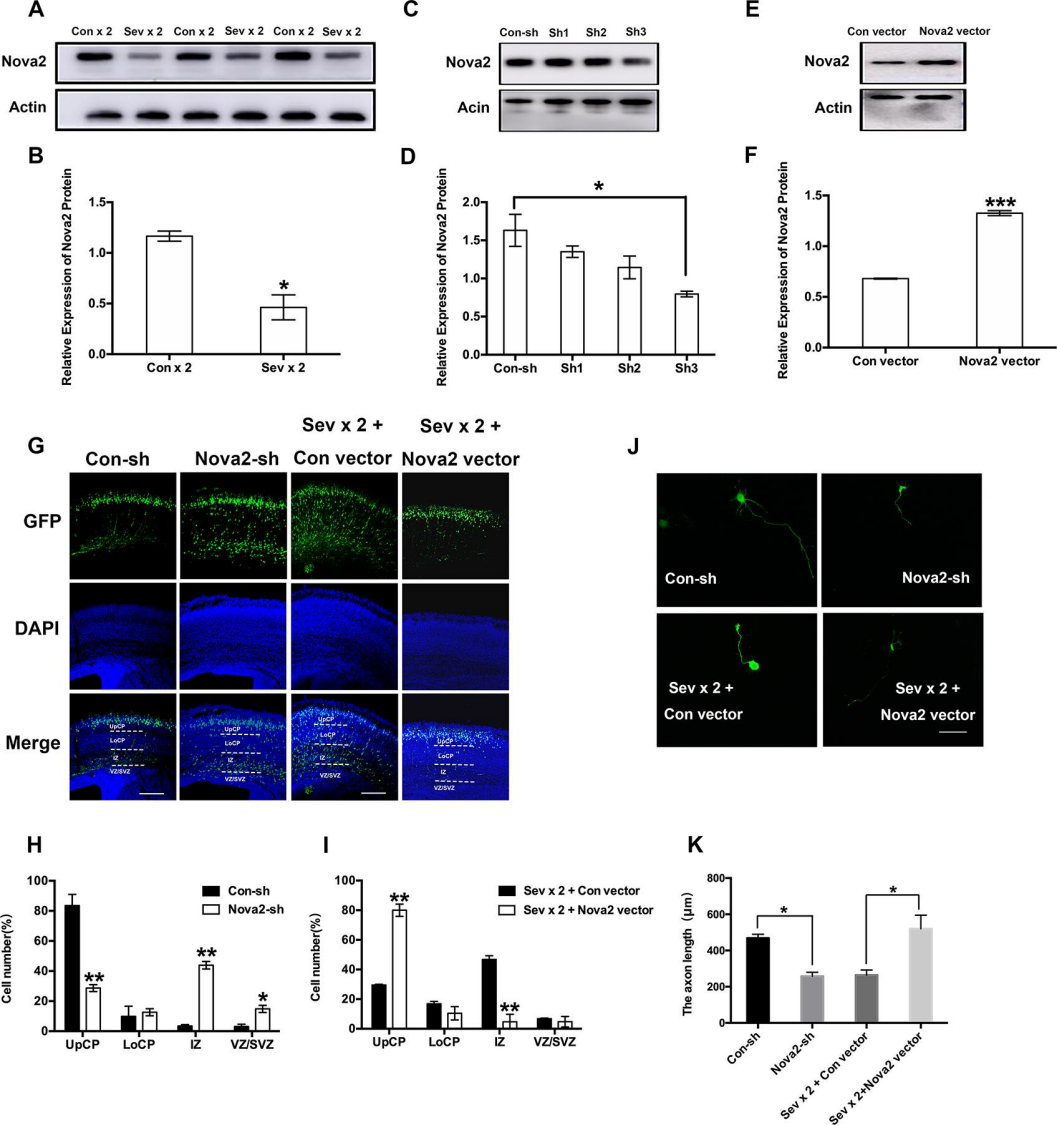
**Nova2 deficiency contributes to the neurotoxicity induced by dual sevoflurane exposure.** (**A**) Western blot analysis demonstrated that dual exposure to sevoflurane decreased Nova2 expression in the cortical tissues of offspring mice. (**B**) Quantification of the protein expressions of Nova2 relative to Actin (*P* = 0.0174*, N = 3, Student’s t-test). (**C**) Nova2 shRNA (sh3) reliably reduces the Nova2 expression. (**D**) Quantification of the protein expressions of Nova2 relative to Actin (*F* = 13.54, *P* = 0.0146*, N = 3, one-way ANOVA). (**E**) Nova2 vector significantly increased the Nova2 expression. (**F**) Quantification of the protein expressions of Nova2 relative to Actin (*P* = 0.0007***, N = 3, Student’s t-test). (**G**) Nova2 knockdown significantly decreased the neuronal migration cortex of offspring mice, while overexpression (OE) of Nova2 reversed loss of function. Scale bars=200 μm. (**H**) Quantification of GFP+ cells at different positions in different groups. Compared to the Con-sh group, the Nova2-sh group had significantly larger fractions of neurons in the IZ (43.86 ± 2.49%, *P* = 0.0021**, N = 3, Student’s t-test) and the VZ/SVZ (14.84 ± 2.4 %, *P* = 0.0283*, N = 3, Student’s t-test), and a significantly smaller fraction of neurons in the UpCP (28.68 ± 2.2768%, *P* = 0.0098**, N = 3, Student’s t-test). (**I**) Quantification of GFP+ cells at different positions in different groups. Nova2 OE reversed dual sevoflurane-induced neuronal migration deficits, neurons primarily migrated out of the IZ (4.735 ± 5.084%, *P* = 0.009**, N = 3, Student’s t-test), and were positioned within the UpCP (80.015 ± 4.1507%, *P* = 0.0034**, N = 3, Student’s t-test) and LoCP (10.47 ± 4.483%, *P* = 0.1957, N = 3, Student’s t-test). (**J**) Nova2 knockdown decreased axon length in primary cultured mouse cortical neurons (*P* = 0.0106*, Student’s t-test), while Nova2 overexpression reversed dual sevoflurane-induced axon growth deficits (*P* = 0.0439*, Student’s t-test). (**K**) The statistical results for the axon length in different groups. Scale bars = 100 μm. *P<0.05. **P<0.01, ***P<0.001.****P < 0.0001.

Western blotting was used to measure cortical Nova2 protein levels in protein lysates collected from embryonic mouse brains. Interestingly, Nova2 gene expression was reduced in the neonatal brain after dual sevoflurane exposure. (Figure 2A, 2B, *P* = 0.01, N = 3, Student’s t-test). However, there were no differences in Nova2 expression between the long-term sevoflurane exposure group and the control group (Supplementary Figure 2A, 2B). Additionally, dual administration of propofol or ketamine, which are intravenous anesthetics, had no effect on Nova2 expression. (Supplementary Figure 2C, 2D). The decrease in Nova2 expression observed after dual sevoflurane exposure might have contributed to inhibited neuronal migration and shorter axon lengths in the developing brain.

To further study the role of Nova2 in neural development, we designed a series of shRNAs against the mouse Nova2 gene and Nova2 vectors. We identified one set of shRNAs that significantly decreased Nova2 protein levels (Figure 2C, 2D, *P* = 0.0146, N = 3, one-way ANOVA) and a Nova2 vector that significantly overexpressed Nova2 (Figure 2E, 2F, *P* = 0.0007, N = 3, Student’s t-test) in mouse primary cortical neurons. We then investigated the role of Nova2 in neuronal migration using in utero electroporation. Plasmids encoding GFP were injected together with Nova2 shRNA into the lateral ventricle. Interestingly, Nova2 knockdown strongly repressed neuronal migration in the fetal brain (Figure 2G and 2H). Compared to the Con-sh group, the Nova2-sh group had significantly larger fractions of neurons in the intermediate zone (IZ) (43.86 ± 2.49%, *P* = 0.0021, N = 3, Student’s t-test) and ventricular zone (VZ)/subventricular zone (SVZ) of the cortex (14.84 ± 2.4%, *P* = 0.0283, N = 3, Student’s t-test), and a significantly smaller fraction of neurons in the upper cortical plate (UpCP) (28.68 ± 2.2768%, *P* = 0.0098, N = 3, Student’s t-test), further indicating that changes in Nova2 protein can interfere with proper neuronal migration. Next, we conducted rescue assays to confirm the role of Nova2 in neuronal migration. Overexpression (OE) of Nova2 successfully reversed loss of function; Nova2 OE neurons exhibited phenotypes similar to those of neurons that only expressed the con vector (Figures 2G and 2I) and were primarily located within the UpCP (80.015 ± 4.1507%, *P* = 0.0034, N = 3, Student’s t-test). In addition, Nova2 knockdown inhibited axons growth to a similar degree as dual sevoflurane exposure (Figure 2J and 2K, *P* = 0.0106, Student’s t-test), while Nova2 overexpression reversed dual sevoflurane-induced axon growth deficits (Figures 2J and 2K, *P* = 0.0439, Student’s t-test).

### Nova2 deficiency suppressed Netrin-1/Dcc activity in the fetal brain

**Figure 3:**
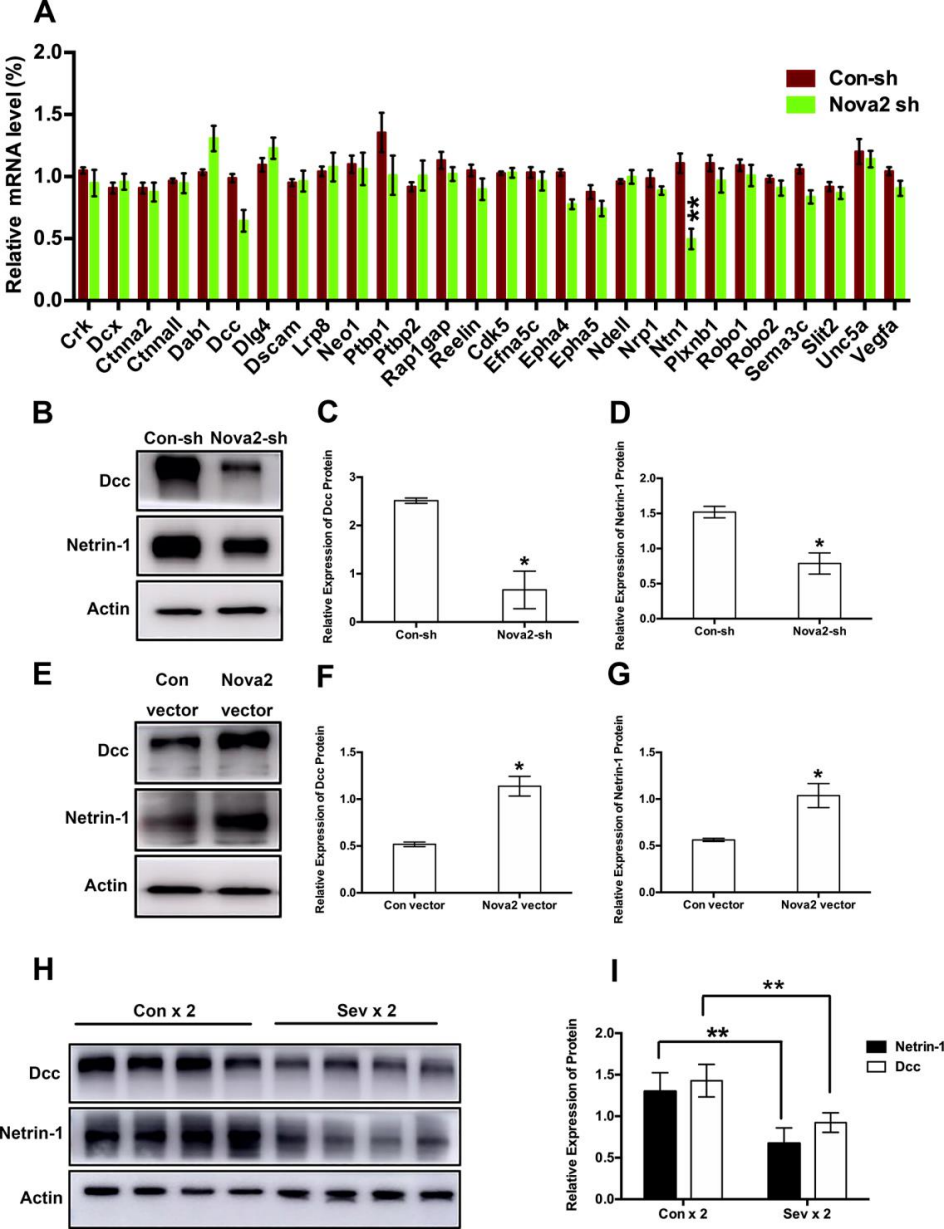
**Both Nova2 knockdown and dual sevoflurane exposure suppressed Netrin-1/Dcc protein expression.** (**A**) QPCR revealed that Netrin-1 mRNA expression was decreased among 28 candidate genes in the Nova2 shRNA group (*F* = 0.76, *P* = 0.0015**, N = 3, Student’s t-test), while Dcc mRNA expression wasn’t change (F = 0.699, *P* = 0.0519, N = 3, Student’s t-test). (**B**) Western blot analysis demonstrated that Nova2 knockdown suppressed Dcc and Netrin-1 proteins expressions. (**C**) Quantification of the protein expression of Dcc relative to Actin (*P* = 0.0217*, N = 3, Student’s t-test). (**D**) Quantification of the protein expression of Netrin-1 relative to Actin (*P* = 0.0264*, N = 3, Student’s t-test). (**E**) Western blot analysis demonstrated that Nova2 OE upregulated Dcc and Netrin-1 protein expressions in the cortical tissues of offspring mice. (**F**) Quantification of the protein expression of Dcc relative to Actin (*P* = 0.0148*, N = 3, Student’s t-test). (**G**) Quantification of the protein expression of Netrin-1 relative to Actin (*P* = 0.0353*, N = 3, Student’s t-test). (**H**) Western blot analysis demonstrated that dual sevoflurane exposure also decreased Dcc and Netrin-1 proteins expressions. (**I**) Quantification of the protein expressions of Dcc (*F* = 1.118, *P* = 0.0044**, N = 3, Student’s t-test) and Netrin-1 (*F* = 0.386, *P* = 0.0052**, N = 3, Student’s t-test) relative to Actin. *P<0.05. **P<0.01, ***P<0.001.****P < 0.0001.

**Figure 4:**
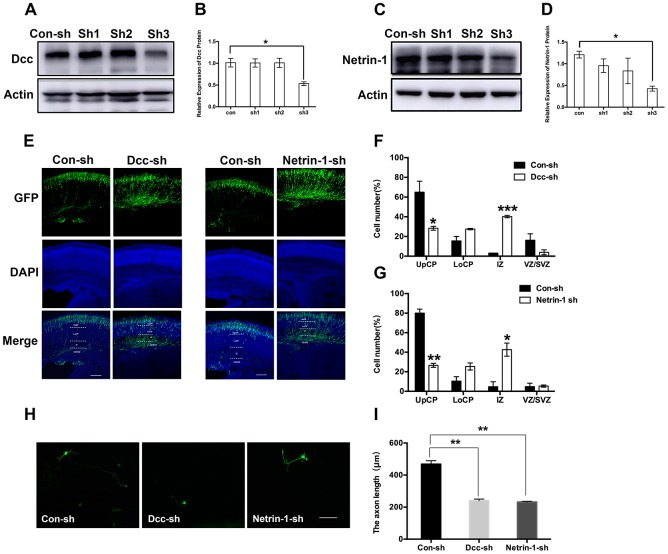
**Netrin-1/Dcc deficiency displayed a similar defect in neuronal migration and axon outgrowth as Nova2 deficiency induced by dual sevoflurane exposure.** (**A**) Dcc shRNA (sh3) reliably reduces the Dcc expression. (**B**) Quantification of the protein expressions of Dcc relative to Actin (*F* = 10.57, *P* = 0.0226*, N = 3, one-way ANOVA). (**C**) Netrin-1 shRNA (sh3) reliably reduces the Netrin-1 expression. (**D**) Quantification of the protein expressions of Netrin-1 relative to Actin (*F* = 7.084, *P* = 0.0445*, N = 3, one-way ANOVA. (**E**) Dcc and Netrin-1 knockdown both significantly inhibited neuronal migration cortex of offspring mice. (**F**) Quantification of GFP+ cells at different positions in different groups. Compared to the Con-sh group, the Dcc-sh group had significantly larger fractions of neurons in the IZ (40.23 ± 1.20208%, *P* = 0.0005***, N = 3, Student’s t-test) and the VZ/SVZ (3.93 ± 2.50315%, *P* = 0.13, N = 3, Student’s t-test), and a significantly smaller fraction of neurons in the UpCP (28.335 ± 1.9728%, *P* = 0.0434*, N = 3, Student’s t-test). (**G**) Quantification of GFP+ cells at different positions in different groups. Compared to the Con-sh group, the Netrin-1-sh group had significantly larger fractions of neurons in the IZ (42.665 ± 6.73872%, *P* = 0.0239*, N = 3, Student’s t-test) and the VZ/SVZ (5.335 ± 1.0818%, *P* = 0.8521, N = 3, Student’s t-test), and a significantly smaller fraction of neurons in the UpCP (26.57 ± 2.0223%, *P* = 0.0037**, N = 3, Student’s t-test). (**H**) Both Dcc and Netrin-1 knockdown decreased axon length of neurons in primary cultured mouse cortical neurons. (**I**) The statistical results for the axon length in Dcc-sh RNA group (*P* = 0.0054**, Student’s t-test) and Netrin-1-shRNA group (*P* = 0.0044**, Student’s t-test). Scale bars = 100 μm; approximately 70 cells from three independent experiments were counted during the statistical analysis. *P<0.05. **P<0.01, ***P<0.001.****P< 0.0001.

**Figure 5:**
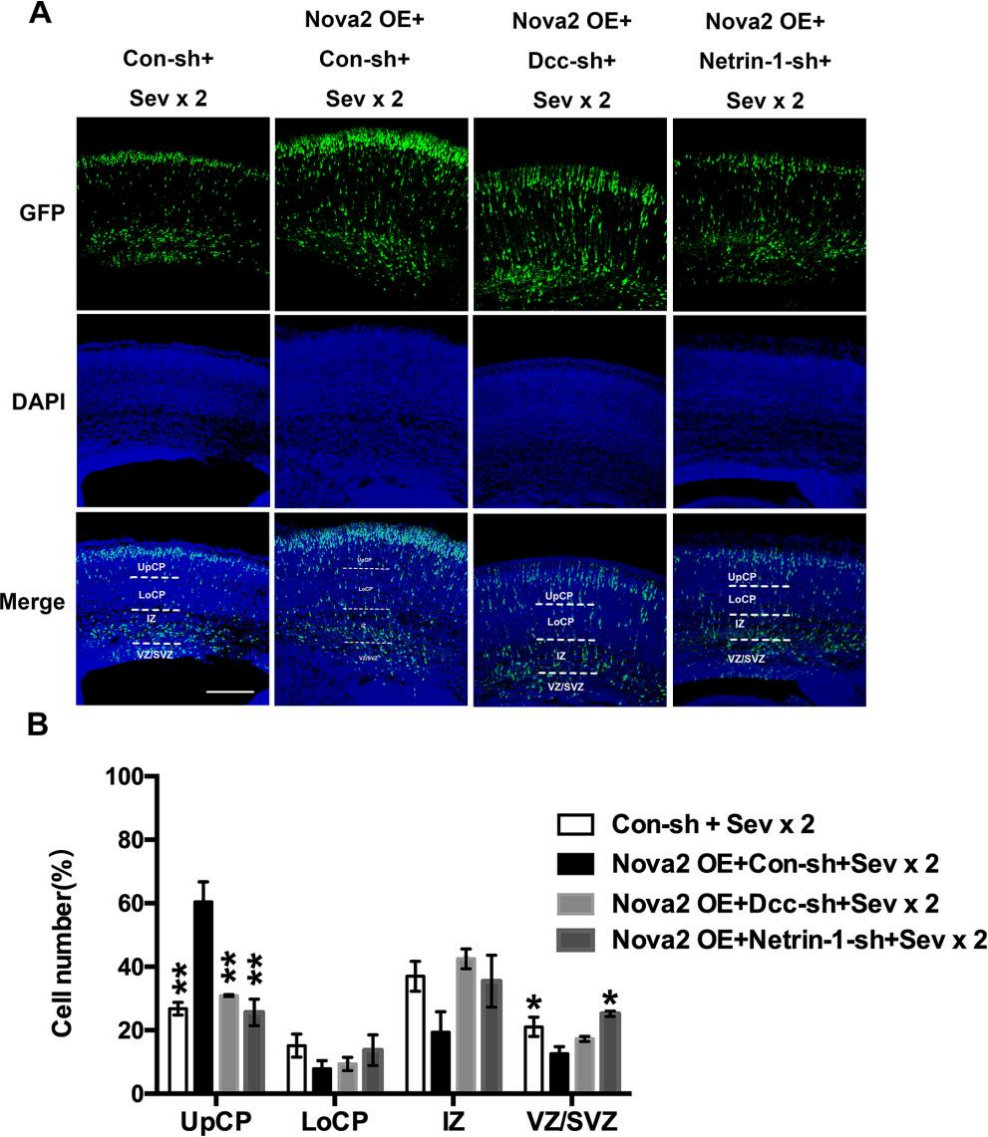
**Nova2 regulates neuronal migration through modulating Netrin-1/Dcc activity in the developing mouse cerebral cortex.** (**A**) Dual sevoflurane exposure significantly decreased neuronal migration cortex of offspring mice, and Nova2 overexpression can rescue the neuronal migration deficits induced by dual sevoflurane exposure. While Netrin-1/Dcc shRNA mitigated the Nova2 rescue phenomenon, which was similar to the neuronal migration deficits induced by dual sevoflurane exposure. (**B**) Quantification of GFP+ cells at different positions in different groups. Similar with dual sevoflurane exposure group, Netrin-1/Dcc knockdown mitigated the Nova2 rescue phenomenon, larger fractions of neurons in the IZ (*F* = 5.672, *P* = 0.0634, N = 3, one-way ANOVA) and the VZ/SVZ (*F* =14.68, *P* = 0.0126*, N = 3, one-way ANOVA), and a significantly smaller fraction of neurons in the UpCP (*F* = 35.79, *P* = 0.0024**, one-way ANOVA). *P<0.05. **P<0.01, ***P<0.001.****P < 0.0001.

To examine the mechanisms underlying sevoflurane-induced neurotoxicity, we conducted a literature search to identify downstream targets of Nova2 that were significantly enriched in sevoflurane-induced neurotoxicity models. In utero electroporation was used to induce uptake of shRNA constructs that resulted in acute Nova2 knockdown in the developing mouse embryo cortex. Of the 28 candidate genes examined in this experiment, Nova2 knockdown decreased Netrin-1 (Ntn1) mRNA expression (Figure 3A, *P* = 0.0015, Student’s t-test). Netrin-1, also known as Ntn1, a ligand for the Deleted in Colorectal Cancer (Dcc) receptor belonging to the laminin-related Netrin family of secreted factors, was first identified as a guidance cue for migrating neuronal progenitors and axons in vertebrate’s nervous system development [22]. Dcc, an Ig family receptor, contains a cytoplasmic region with three conserved domains, namely P1, P2, and P3. Despite advances in the study of Netrin-1/Dcc signaling, little is known about how the intracellular Dcc signaling complex is organized or about associated cellular signal transduction mechanisms. Several recent *in vitro* studies indicate that the Nova2 may regulate alternative Dcc splicing during neuronal migration and axon guidance in the spinal cord [21]. However, there is no direct evidence of a relationship between Nova2 and the Netrin-1/Dcc receptor. It is therefore unclear whether and how Netrin-1/Dcc acts as a downstream effector of Nova2 signaling. First, we examined the effects of Nova2 expression on Dcc and Netrin-1 protein levels in the fetal brain. Western blotting revealed that Nova2 knockdown (Nova2 shRNA) suppressed Dcc and Netrin-1 expression (Figure 3B, Figure 3C (*P* = 0.0217, N = 3, Student’s t-test), Figure 3D (*P* = 0.0264, N = 3, Student’s t-test)), while overexpression of Nova2 (Nova2 vector) upregulated Dcc and Netrin-1 levels (Figure 3E–3G, Figure 3F (*P* = 0.0148, N = 3, Student’s t-test), Figure 3G (*P* = 0.0353, N = 3, Student’s t-test)). Furthermore, Nova2 directly interacted with Netrin-1/Dcc in the cerebral cortex (Supplementary Figure 3). We then explored whether dual sevoflurane exposure affected the expression of these two proteins. Indeed, levels of both proteins were also decreased in the neonatal brain after dual sevoflurane exposure in the second trimester (Figure 3H, 3I). Although previous studies have shown that Nova2 deficiency may disrupt Netrin-induced ventral projection to the midline and axon outgrowth [21], there is no evidence that Netrin-1/Dcc have adverse effects on neuronal migration or axon length during neonatal brain development. We therefore developed a set of shRNAs that significantly decreased Dcc protein (Figure 4A and 4B, *F* = 10.57, *P* = 0.0226, N = 3, one-way ANOVA) and Netrin-1 protein (Figure 4C and 4D, *F* = 7.084, *P* = 0.0445, N = 3, one-way ANOVA) levels. We demonstrated for the first time that Netrin-1/Dcc knockdown similarly inhibited neuronal migration and axon length (Figure 4E–4H). Finally, we found that Netrin-1/Dcc knockdown mitigated the Nova2-induced reversal of deficits in neuronal migration after dual sevoflurane exposure (Figure 5A, 5B).

## DISCUSSION

Neurotoxicity in fetuses following maternal exposure to sevoflurane may result in neurodevelopmental impairments. Recent studies have shown that use of anesthetic drugs during the critical rapid growth phase of early brain development can cause neurodegenerative changes and impaired learning in neonatal animals [26–28]. For example, a single four-hour exposure to 1.4% isoflurane (1.3 minimum alveolar concentration) during mid-gestation led to long-term spatial memory impairment in rat offspring [29, 30]. We previously found that dual sevoflurane exposure can lead to cognitive dysfunction in young animals, while single short (two-hour) or long (six-hour) duration exposure didn’t affect spatial learning (data not shown), suggesting that fetuses were particularly vulnerable to dual exposure.

An understanding of the molecular mechanisms that contribute to fetal neurotoxicity and neuronal development is important for guiding decisions about the administration of anesthesia during pregnancy. Migration of neurons is crucial for structural organization of the cerebral cortex [4], and abnormal neuronal migration in perinatal children can lead to various cerebral cortical developmental disorders, such as focal cortical dysplasia, nodular sclerosis, microcephaly, gray matter heterotopia, and lissencephaly [5]. Dysregulation of neuronal migration may also contribute to abnormal development in neonates if the pregnant mother undergoes an operation requiring anesthesia during the second trimester.

In contrast, we found that a single six-hour exposure to sevoflurane and dual two-hour intravenous exposure to propofol or ketamine had no significant effects on neuron migration or axon length, suggesting that intravenous anesthetics can be used safely in pregnancy. In comparison, dual two-hour exposures to sevoflurane may cause neuronal migration deficits and reduce axon length, which in turn perturbs normal development of the central nervous system and may result in learning and memory deficits in offspring. These results indicate that dual sevoflurane exposure should be avoided during pregnancy if possible.

Decreases in the number of neurons in the cortical plate and aberrant migration of Purkinje neurons were detected in mice with Nova2 deficiency using exon junction arrays [31] and HITS-CLIP [14]. Reduced Nova2 expression in mice causes developmental defects and neonatal lethality [18–19]. Nova2 knockdown-induced decreases in the number of neurons that arise from the margins of the embryonic cerebral ventricles and migrating to the marginal zone and cortical plate likely reflect a change postmitotic neuron migration rather than changes in neural progenitor cell fate [20]. Such changes in fetal nervous systems development also play an important role in lamination in the brain.

Here, we found that Nova2 expression in the brain is reduced in neonatal mice after pregnant mice are exposed to sevoflurane twice (Figure 2). These changes in Nova2 expression may cause changes in neuronal migration and axon outgrowth. Gene knockdown studies demonstrated that Nova2 knockdown in the brain increased the number of GFP-positive neurons within the VZ, indicating abnormalities in neural migration patterns. In addition, axons were shorter, suggesting a defect in axon growth and/or guidance, after dual sevoflurane exposure; such axons may fail to reach the midline, (Figure 3).

Nevertheless, the mechanisms by which Nova2 proteins suppress neuronal migration remain unclear. Previous studies have reported significant changes in Dcc exon 17 in Nova2^-/-^ mice [13]. Dcc plays various roles in the nervous system, including modulating dendritic growth and guidance [32–35] and synapse formation and function [36–38]. Consistent with previous studies, we found that Dcc protein levels decreased when Nova2 expression was inhibited. Interestingly, we demonstrated that Netrin-1 protein levels decreased dramatically as well. Netrins are a family of proteins that direct cell and axon migration during development. Receptors for secreted netrins include Dcc and the UNC5 homologs in mammals. Dcc mediates chemo-attraction, while repulsion requires a UNC5 homolog. Netrin-1-Dcc signaling is required to stimulate commissural axon outgrowth and to attract axons to the ventral midline [21]. When comparing the effects of Netrin-1/Dcc knockdown and Nova2 knockdown, we found that knockdown of either Netrin-1 or Dcc increased numbers of GFP+ neurons within the SVZ/VZ and IZ similarly to Nova2 knockdown. Additionally, as was the case for Nova2, knockdown of either Netrin-1 or Dcc reduced axon lengths (Figure 4). CO-IP suggested that Nova2/Netrin-1/Dcc may exert their effects by forming a complex. While knockdown of all three factors results in similar defects in neuronal migration, axon outgrowth, and axon guidance, whether these defects are temporary or persist for a long time remains unknown. Nova2 proteins may also play a part in other pathological processes, such as abnormal methylation or alteration of synaptic properties [39]. Moreover, Dcc alternative splicing is perturbed by Nova2 deficiency *in vivo* [21], and alternative splicing might therefore be involved in sevoflurane-induced deficits in neuronal development. Finally, Nova2 proteins might also have various other biochemical effects, including alternative mRNA polyadenylation [14] and functional regulation of Argonaute-microRNA complexes [40]. Further studies are necessary to fully characterize the role of Nova2 during neural development.

In conclusion, we demonstrated here that dual sevoflurane exposure-induced decreases in Nova2 expression resulted in neuronal migration deficits in the fetal brain that eventually led to learning and behavioral deficits. In addition, our data suggested that Netrin-1/Dcc might be an effective target for drugs designed to decrease anesthetic absorption and protect fetal development if anesthesia is necessary during pregnancy. Further study of clinical anesthetics and their effects on neuronal migration may provide new methods for preventing and treating anesthesia-induced fetal neurotoxicity.

## MATERIALS AND METHODS

### Animals and anesthetic procedures

All experiments were conducted in compliance with the ARRIVE guidelines. Pregnant C57BL/6 mice at embryonic day 14.5 (E 14.5) were obtained from the SLAC Laboratory Animal Co., Ltd. (Shanghai, China). All animals were fed a standard diet and were housed at 22 °C with a 12 h light–dark cycle. The study protocol was reviewed and approved by the Bioethics Committee of the Institute of Neuroscience at the Chinese Academy of Sciences.

Gas inhalation anesthesia was administered in a specific cage with mixed gas, including oxygen, air, and sevoflurane. Two ducts with diameters of 8 mm were connected to two different walls: one duct was used for gas delivery, and the other was used for continuous monitoring of the gas sample. A specific vaporizer (Penlon Ltd., Abingdon, UK) was used to administer sevoflurane. In addition, an infrared detector (Datex-Ohmeda, Helsinki, Finland) was used to detect the concentration of sevoflurane in the mixed gas. Mice in the Sev x 2 group received 2.5% sevoflurane in 60% oxygen on two occasions, while mice in the Con x 2 group received 60% oxygen without sevoflurane. In a pilot study, we found that this sevoflurane administration regimen didn’t change arterial blood gas or pH values in our current study (Table 1). A heating mat warmed to approximately 28 °C was used to maintain room temperature inside the cage (~22–24 °C). After anesthesia, the animals were transferred to normal housing cages with normal air.

**Table 1:** Blood Gas and Blood Glucose Analyses during Gas Exposure.

**Group**	**Time point**	**pH**	**PaO_2_ mmHg**	**PaCO_2_ mmHg**	**Hct %**	**Glucose mg/dl**
60% O_2_	Before exposure	7.33±0.04	142.5±6.8	33.1±4.2	32.4±3.7	71.2±13.2
	2h in 1^st^ exposure	7.34±0.03	148.9±9.5	34.7±4.8	34.8±3.9	76.9±11.2
	2h in 2^nd^ exposure	7.35±0.02	155.0±11.2	30.4±5.0	35.8±4.2	77.8±10.2
2.5% Sev	Before exposure	7.32±0.05	153.5±10.8	35.2±3.6	32.8±5.1	70.8±11.2
	2h in 1^st^ exposure	7.33±0.08	149.2±8.1	34.2±4.6	34.2±6.3	71.5±14.7
	2h in 2^nd^ exposure	7.31±0.06	138.2±7.3	37.8±4.7	35.2±5.8	76.9±13.3

Hct: hematocrit; Sev: sevoflurane; PaCO_2_: arterial carbon dioxide tension; PaO_2_: arterial oxygen tension. *P<0.05 v.s. before exposure group. Data are presented as means ± standard deviation (SD). (n=5).

At E18.5, the pregnant mice in each group underwent cesarean sections under deep anesthesia via an intraperitoneal injection of 0.7% sodium pentobarbital (10 mL/kg) to deliver pups. Whole brains were immediately harvested from the pups, frozen in liquid nitrogen, and stored at -80 °C.

### Plasmids and constructs

Nova2, Netrin-1, and Dcc shRNA sequences were synthesized as oligonucleotide primers and manually annealed. The following primers were utilized:

**Table:** 

Nova2 oligo forward:	5’-CTAGAGGAACCGGCGGGTCACCATCATTCAAGAGATGATGGTGACCCG CCGGTTCCTTTTTTG -3’
Nova2 oligo reverse:	5’-GATCCAAAAAAGGAACCGGCGGGTCACCATCATCTCTTGAATGATGGT GACCCGCCGGTTCCT-3’
Dcc oligo forward:	CTAGAGCCATGACAGTCAATGGTACTTTCAAGAGAAGTACCATTGACT GTCATGGCTTTTTTG -3’
Dcc oligo reverse:	5’-GATCCAAAAAAGCCATGACAGTCAATGGTACTTCTCTTGAAAGTACCA TTGACTGTCATGGCT -3’
Netrin-1 oligo forward:	5’-CTAGAGGAAGTTCACCGTGAACATCATTCAAGAGATGATGTTCACGGT GAACTTCCTTTTTTG-3’
Netrin-1 oligo reverse:	5’-GATCCAAAAAAGGAAGTTCACCGTGAACATCATCTCTTGAATGATGTTC ACGGTGAACTTCCT-3’
Scrambled shRNA oligo forward:	5’-gatctGTTCTCCGAACGTGTCACGTTTCAAGAGAACGTGACACGTTCGGAG AATTTTTTc-3’
Scrambled shRNA oligo reverse:	5’-aattgAAAAAATTCTCCGAACGTGTCACGTTCTCTTGAAACGTGACACGTT CGGAGAACa-3’

The Nova2, Netrin-1, Dcc, or control shRNA sequences were inserted into FUGW-H1-GFP vectors [41] (Addgene, 37632) to create corresponding shRNA plasmids.

**Table:** 

Nova2 forward:	5’-CCAAGCAGGCCAAGCTCATCGTCCCC-3’
Nova2 reverse:	5’-AACATCGTATGGGTATCCCACTTTCTGTGGGTTTGAAGCC -3’
Dcc forward:	5’-ACTCCGCAGCTTTGCTAACCCATTACTACCTCCACCCATGAG-3’
Dcc reverse:	5’-CTGCCCTCTCCACTGCTAGCGTAATCTGGAACATCGTATGGGT-3’
Netrin-1 forward:	5’-GGAAATTTACTGTCAATATTATCTCCGTGTACAAGCAGGGCACA-3’
Netrin-1 reverse:	5’-ACATCGTATGGGTAGGATCCGGCCTTCTTGCACTTGCCCTTCT -3’

The Nova2, Netrin-1, Dcc sequences were inserted into FUGW-2A vectors to create Nova2, Netrin-1, and Dcc overexpression plasmids.

### In utero electroporation (IUE)

Plasmids were prepared in a final volume of 15 μL (2 μg/μL plasmid DNA, 0.01% Fast Green, and 0.5 μg/μL enhanced GFP) for each pregnant mouse. The plasmids were then injected into the ventricles of the E14.5 mouse brains. Electric pulses were produced with a T830 electroporator (BTX Molecular Delivery Systems, Holliston, MA, USA) and applied in five 30 V, 50 ms pulses with an interval of 1 s for each embryo. After perfusion, brains were selected under a fluorescent dissecting microscope (Nikon 80i, MBA75020, Japan); only those brains with EGFP expression were used for further immunofluorescence staining.

Mouse pups were harvested at E 18.5, and whole brains were fixed in 4% PFA for 24 h and sequentially dehydrated in 15% and 30% sucrose solutions (in 1 x PBS) for 48 h. Longitudinal brain sections (30 μm) were obtained via frozen sectioning for later immunofluorescence experiments in which GFP immunostaining was performed using a GFP antibody and an Alexa 488-conjugated secondary antibody to recognize GFP positive neurons. The detailed experimental protocol is shown in Supplementary Figure 1A.

### Cell culture and transfection

Embryonic day 14.5 (E14.5) mouse cortical neurons were cultured and transfected with the plasmids described above via electroporation with an Amaxa Nucleofector (Amaxa, Cologne, Germany) on the first day of *in vitro* (DIV) culture on cover glasses coated with a Poly-D-lysine solution according to the manufacturer’s instructions. After DIV 3, the cells were fixed for further immunofluorescence analysis. The detailed neuron electroporation experimental protocol is shown in Figure 1A.

### Morris water maze

The spatial learning and memory abilities of the mice were evaluated during MWM training and probe tests. MWM testing was conducted in a round white pool 94 cm in diameter and 45 cm deep. The pool was filled to a depth of 30 cm with water made opaque with white nontoxic water-based tempura paint. Pool temperature was maintained at 22 ± 1 °C. The pool was artificially divided into four conceptual quadrants (NE, SE, NW, and SW). A platform (20 cm diameter) was submerged 1 cm below the water surface in the center of the north-west quadrant of the pool. For probe trials, the platform was removed. Four distal extra-maze cues (a traffic cone, a colorful poster, and two black-and-white construction paper designs) were placed around the pool and remained in the same positions throughout the training and testing periods. The swim path was recorded on a camera mounted above the center of the pool, and a video racking motion analysis system was used to track movement (Ethovision, Noldus, version 4.1). The MWM was illuminated by 75 lx and surrounded by white curtains on which the distal cues were located. The animals were trained in the MWM four times a day for four consecutive days. In each training session, the animals were released into one of the four imaginary quadrants. The animals were allowed to swim for 60 s or until they reached the escape platform. When the animals reached the platform, they were removed from the water after remaining on the platform for 15 s. During training, if the animals could not find the platform during the first 60 s, they were directed by hand and left on the platform for 15 s to provide spatial information about the location of the platform. The platform remained in the same quadrant during all tests. After removal from the pool, mice were manually dried with a terrycloth towel and placed in a warming cage consisting of a heating pad set to low underneath a typical shoebox cage for at least 5 min before returning to the home cage. The animals were tested on day 5. At the time of the test, the platform was removed from the water, and the animals were placed in the quadrant opposite the previous location of the platform. The animals were allowed to swim for 60 s during the test. Time elapsed before reaching the previous platform location (escape latency), time spent in the quadrant where the platform had been (seconds, s), the number of times the previous platform location was crossed, and swimming speed were recorded. The detailed experimental protocol is shown in Figure 1B.

### RNA extraction and real-time PCR

Total RNA was extracted using an RNeasy mini kit (QIAGEN, Hamburg, Germany). For real-time PCR, RNA (2 mg) was reverse transcribed to generate complementary template DNA (40 ng). The amplification cycling reactions (40 cycles) conditions were as follows: 5 s at 95 °C and 30 s at 60 °C. Data analysis was completed using the comparative ΔΔCT method in QuantStudio 6 Flex software. Measurement of precursor and mature miRNA levels were conducted as described previously.

Primers used in quantitative real-time PCR assays were as follows:

**Table:** 

Nova2 forward:	5’-GGCCTCATCCCACTTTCTGTG -3’
Nova2 reverse:	5’-AAGCCGCTCAATACCTCATCA-3’
GAPDH forward:	5’-AAGAAGGTGGTGAAGCAGG-3’
GAPDH reverse:	5’-GAAGGTGGAAGAGTGGGAGT-3’
Reelin forward:	5’-ATGTTGTTCTCACTGCCGATA-3’
Reelin reverse:	5’-GAGATCAAGCCTGACTTATGG-3’
Dab1 forward:	5’-GCTTATCCTTTTGTGCCTTTT-3’
Dab1 reverse:	5’-CCTGTTATCCTGGACTTGAGA-3’
Dcc forward:	5’-GTTCCCGTAGGCTTCTCG-3’
Dcc reverse:	5’-CAACCTGCTTGTAATAACCG-3’
Dcx forward:	5’-CCAGTTGGGGTTGACATTCTT-3’
Dcx reverse:	5’-CGCTGTTTCTTCTGACCGTTTT-3’
Ndell forward:	5’-TTGCGGGATGCTTGGTCTTTT-3’
Ndell reverse:	5’-TTCTGCGGTCCAGGCTTCACT-3’
Robo1 forward:	5’-GCTTCGTCCTCCTCCTCTTCT-3’
Robo1 reverse:	5’-TTCCCCACCTCATACTTACGG-3’
Robo2 forward:	5’-GGTAGTCAGGGATGTAAAGTAA- 3’
Robo2 reverse:	5’- GCCATTTATAGCAGCATTG- 3’
Ptbp1 forward:	5’- AATTCCCAGCACCTGCCAACC- 3’
Ptbp1 reverse:	5’- TGGAGACAGCCAGCCTTCACT- 3’
Ptbp2 forward:	5’- ACCAAAAGGTAAACCTAAAGC- 3’
Ptbp2 reverse:	5’- CCAACGGTAACGATAGTAAGA- 3’
Ctnnall forward:	5’- GGTTTTCCTTAGCGATTTTGC- 3’
Ctnnall reverse:	5’- GCTTTGGCTGAGTATGCCTGTA- 3’
Lrp8 forward:	5’- CAGCATCTTTCTGTTGCCTCC- 3’
Lrp8 reverse:	5’- ACTGGTCTGACTGGGGTTTCC- 3’
Crk forward:	5’- CAGTAAAGCAGGCAATGAATC- 3’
Crk reverse:	5’- CGTCTCCCACTACATCATCAA- 3’
Rap1gap forward:	5’- CTCTGACTTGGGCTCTTGTGA- 3’
Rap1gap reverse:	5’- GGAAGAAGTCTGGTCCGTTTG- 3’
Ctnna2 forward:	5’- GGAAGAAGTCTGGTCCGTTTG- 3’
Ctnna2 reverse:	5’- AAGTGCTGGAAGCCACAAAAT- 3’
Cdk5 forward:	5’- CGGAGGGCTGAACTTGGCACA- 3’
Cdk5 reverse:	5’- TGGAGAAGATTGGGGAAGGCACC- 3’
Ntn1 forward:	5’- GGTTATTGAGGTCGGTGAGG- 3’
Ntn1 reverse:	5’- CTTGCTCGGATGAGAATGGA- 3’
Sema3c forward:	5’- GGGTTGAAAGAGCATCGTCCT- 3’
Sema3c reverse:	5’- TATGTCTGTGGGAGTGGAGCG- 3’
Nrp1 forward:	5’- ATAGACCACAGGGCTCACCAG- 3’
Nrp1 reverse:	5’- AATCAGAGTTCCCGACATACG- 3’
Plxnb1 forward:	5’- TGCTCCATCTGGGACCTTGTA- 3’
Plxnb1 reverse:	5’- CTGGGCACCTTATCCTTTCTG- 3’
Dlg4 forward:	5’- GGATGAAGATGGCGATAGGGT- 3’
Dlg4 reverse:	5’- GGGAGAAAATGGAGAAGGACA- 3’
Dscam forward:	5’- TGCCCCACTGCTCACTGTTAT- 3’
Dscam reverse:	5’- TCGGCTCACCGTATCCAAGAC- 3’
Vegfa forward:	5’- TGCTGGCTTTGGTGAGGTTTG- 3’
Vegfa reverse:	5’- GCTACTGCCGTCCGATTGAGA- 3’
Unc5a forward:	5’- GCTGCCGTCAGTGCTGCGTTC- 3’
Unc5a reverse:	5’- CTGCCTGGCTTCGTGGTTCGG- 3’
Efna5 forward:	5’- GGAATCTGGGGTTGCTGCTGT- 3’
Efna5 reverse:	5’- GACGCTGCTCTTTCTGGTGCT- 3’
Epha4 forward:	5’-AAAACATAGGAAGTCAGAGGG- 3’
Epha4 reverse:	5’- CCAGGCTAAAGAAGTTACAAG- 3’
Epha5 forward:	5’- CTTGGGTTTCGTATCAGT- 3’
Epha5 reverse:	5’- TGTAATGTGGGAAGTTGTAT- 3’
Neo1 forward:	5’- GTGATGACGCCAACAGAG- 3’
Neo1 reverse:	5’- AAGGAAGCCGACTACCAG- 3’
Slit2 forward:	5’- TCTCAGAGGTCCACAGCA- 3’
Slit2 reverse:	5’- CAGAAGCAGCAGGGTTAC- 3’

### Antibodies

The following antibodies were used in this study: Nova2 (no. 55002-1-AP; Proteintech, Danvers, MA, USA); Netrin-1(no. ab126729; Abcam, Cambridge, UK); Dcc (no.19123-1-AP; Proteintech, Danvers, MA, USA); Actin (no. ab8245; Abcam, Cambridge, UK); Secondary antibody (no. ab205718; Abcam, Cambridge, UK).

### Immunofluorescence

Cells were washed with 1x phosphate-buffered saline (PBS) for 5 min, fixed in 4% paraformaldehyde (PFA) for 20 min, and blocked in 1 x PBS buffer with 3% bovine serum albumin and 0.1% Triton-X-100 for 2 h at room temperature (RT). The cells were incubated in primary antibodies overnight at 4 °C, washed three times in 1x PBS, and then incubated in secondary antibodies at RT for 90 min. Signals were observed via fluorescence microscopy.

### Immunofluorescence staining

Whole fetal cerebral hemispheric cortexes were harvested at E18.5 and fixed in PFA overnight. Frozen 30 μm-thick sections were used for immunofluorescence staining. Sections were washed in PBS for 15 min, blocked with 5% bovine serum albumin, and treated with 0.3% Triton at 37°C for 1 h. Primary antibodies (anti-GFP, Sigma, 1:1000 dilution) were a1-3dded to the sections followed by incubation overnight at 4°C. The sections were then washed for 30 min and incubated with an Alexa 488-conjugated second antibody (no. ab150077; Abcam, Cambridge, UK, rabbit, 1:1000) at 37°C for 1 h.

### Western blot

Whole fetal cerebral hemispheric cortexes were harvested at E18.5. For Nova2 expression, cortical tissue homogenates were prepared, immersed in RIPA buffer, boiled in 1 x sodium dodecyl sulfate loading buffer, and then resolved by 8% sodium dodecyl sulfate-polyacrylamide gel electrophoresis. Proteins on the gel were then transferred to polyvinylidene fluoride membranes (Amersham Biosciences, Uppsala, Sweden), and the membranes were blocked with 5% nonfat milk in Tris-buffered saline and Tween 20 (TBST) buffer for 1 h. The membranes were then incubated in primary antibodies overnight at 4 °C, washed three times in TBST, and incubated in secondary antibodies for 1 h at RT. Staining was visualized via horseradish peroxidase reactions using SuperSignal Chemiluminescent Substrate (Thermo Fisher Scientific, Waltham, MA, USA).

### Data analysis

Images were obtained using a Nikon A1 fluorescence microscope (Nikon, Tokyo, Japan) equipped with a 20× objective for axon length in cultured mouse cortical neurons and a Nikon A1 equipped with a 10× objective for cortical migration and IUE. To evaluate axon length in cultured primary cortical neurons, GFP positive cells were randomly selected from each condition, and the total axon length was traced using the Fiji plugin for ImageJ software (http://imagej.net/Fiji/Downloads). At least three independent experiments were performed, and approximately 70 neurons were analyzed. For IUE, GFP positive cells were counted using ImageJ software (https://imagej.nih.gov/ij/download.html). The ratios of GFP+ cells in the upper cortical plate (UpCP), lower cortical plate (LoCP), intermediate zone (IZ), subventricular zone (SVZ), and ventricular zone (VZ) of the cortex were calculated and compared between groups. All values are presented as means ± standard deviation (SD). *P* < 0.05 was considered significant.

## Supplementary Material

Supplementary Materials

Supplementary Figures
