# Supplementary material for: Sevoflurane inhibits neuronal migration and axon growth in the developing mouse cerebral cortex
Source: Aging (Albany NY). 2020 Apr 9;12(7):6436–55. doi: 10.18632/aging.103041 (PMC7185136; doi:10.18632/aging.103041)
Supplement: Supplementary Materials [file aging-12-103041-s001..pdf]

SUPPLEMENTARY MATERIALS AND METHODS

Animals and anesthetic procedures

In total, pregnant mice were randomized into 6 groups at embryonic day 14.5: Sev 6 h, Con 6 h, DMSO, Propofol, NaCl, and Ketamine.

Drug injection: All drugs were prepared just before use. On E14.5, pregnant mice received an intravenous infusion of propofol (Diprivan; AstraZeneca UK limited, Italy: jc393, 20 mL: 200 mg) with an equal volume of DMSO at a rate of 60 mg /kg/h for 2 h, for two consecutive days. Also, on E14.5, other pregnant mice received an intravenous infusion of ketamine with an equal volume of Nacl at a rate of 75 mg /kg/h for 2 h, for two consecutive days.

Co-Immunoprecipitation (CO-IP)

The Co-IP experiments were conducted utilizing the Pierce Crosslink IP kit (Thermo Fisher Scientific, Inc.) following the manufacturer's protocol. When plasmids expressing Flag-tagged Nova2 and HA-tagged Netrin-1/His-tagged Dcc were transfected into the ventricles of E14.5 mouse brains by in utero electroporation, brain tissues protein lysate was immunoprecipitated respectively with anti-Flag, anti-HA, anti-His antibodies, and negative control IgG antibody overnight at 4 °C under constant rotation. The brain tissues were washed with precooled PBS, then after blotting with filter paper, the tissues were transferred into a precooled Eppendorf (EP) tube. According to the weight of the cerebral cortex, the tissues were added to precooled Co-IP Lysis Buffer (freshly mixed with protease and phosphatase inhibitor) and placed on ice for 30 min, centrifuged at 12000 g for 20 min, and transferred into a new EP tube, followed by the detection of the protein concentration. One aliquot of the supernatant was saved as the input control, the remainder was incubated with the Protein G agarose beads (no. 11719233001, Roche, Mannheim, Germany). The Protein G agarose beads were rinsed with precooled PBS and then activated three times at 2000 g for 1 min at 4 °C. Every 1 mL total protein was added to 100 μL Protein G agarose beads and oscillated at 4 °C overnight to remove the background. With the addition of primary anti-His, anti-HA, and anti-Flag, the antigen-antibody mixture was slowly oscillated at 4 °C for 4-6 hours and oscillated at 4 °C overnight with the addition of 100 μL activated Protein G agarose beads. After centrifugation at 2000 g for 1 min at 4 °C, the antigen-antibody and the Protein G agarose beads were collected. After the removal of the supernatants, the proteins were washed with precooled Co-IP Lysis Buffer three times (800 μl buffer/time), centrifuged at 2000 g for 1 min at 4 °C, mixed with 60 μl 1.5 × Co-IP loading buffer, and boiled at 100 °C for 10 min. The subsequent procedures were the same as those of the western blot analysis.

Antibodies

The following antibodies were used in this study: Flag (no. 66008-3-Ig; Proteintech, Danvers, MA, USA); HA (no. 66006-2-Ig; Proteintech, Danvers, MA, USA); His (no. 66005-1-Ig; Proteintech, Danvers, MA, USA); and IgG (no. A7016; Beyotime Institute of Biotechnology).
