# Supplementary material for: Sevoflurane inhibits neuronal migration and axon growth in the developing mouse cerebral cortex
Source: Aging (Albany NY). 2020 Apr 9;12(7):6436–55. doi: 10.18632/aging.103041 (PMC7185136; doi:10.18632/aging.103041)
Supplement: Supplementary Figures [file aging-12-103041-s002..pdf]

SUPPLEMENTARY FIGURES

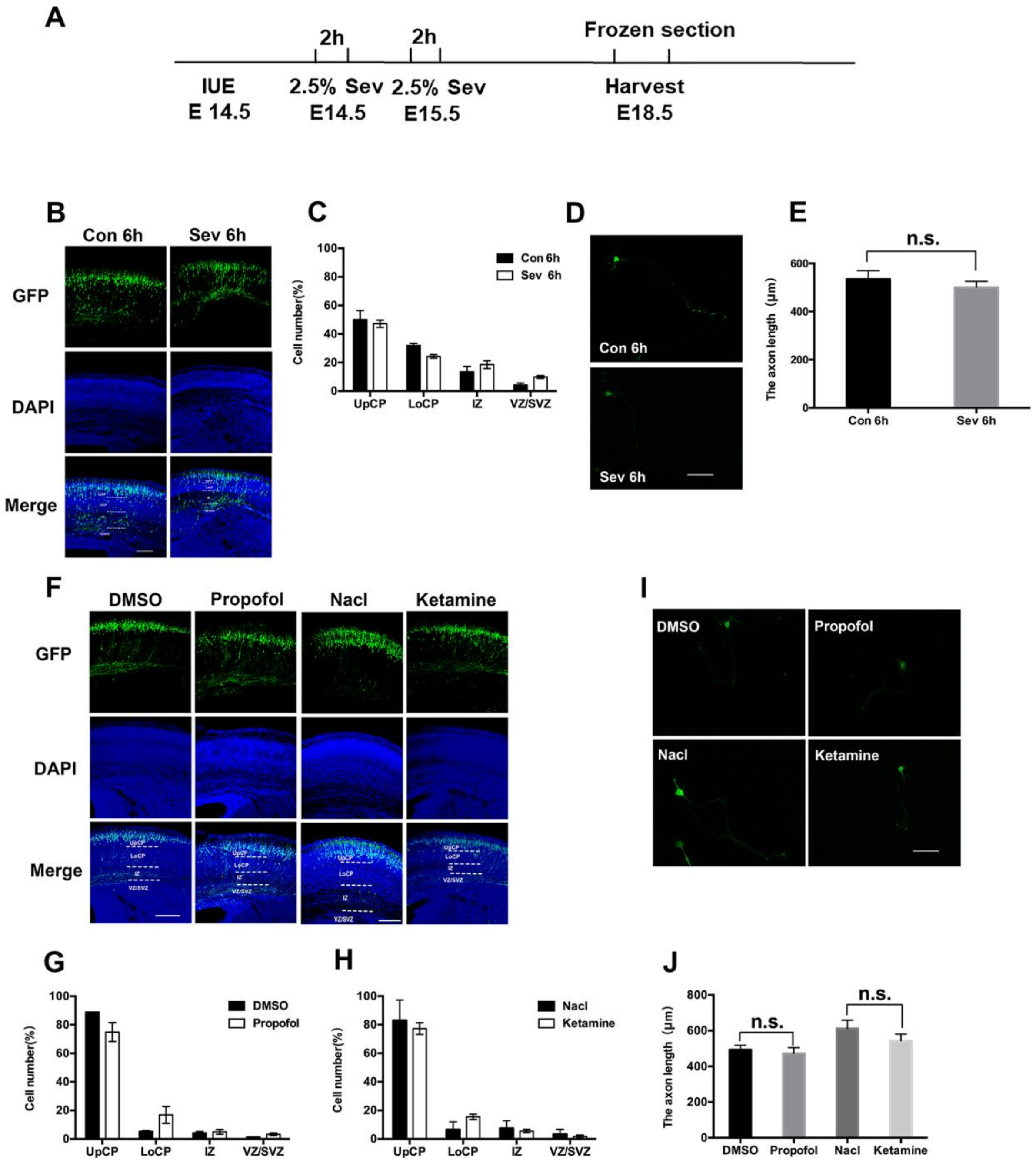

Supplementary Figure 1. Neither single six-hour sevoflurane exposure nor dual two-hour intravenous anesthetics exposure had adverse effect on neuronal migration or axon length. Control vector (Fugw-H1-GFP alone) was transfected in utero on E14.5 pregnant mice in different groups. Representative coronal sections of mouse brains revealed migration of transfected cells. (**A**) Flowchart of the IUE experiment. (**B**) Single six-hour sevoflurane exposure did not affect neuronal migration. All sections were counterstained with DAPI (blue) nuclear counterstain. (**C**) Quantification of GFP+ cells in different positions. The GFP+ cells in the UpCP, LoCP, IZ, and VZ/SVZ were counted, and the ratio of GFP+ cells was analyzed statistically using unpaired t-tests. Scale bars=200 μm. (**D**) Single six-hour sevoflurane exposure did not affect axon length of neurons in primary cultured mouse cortical neurons as well. (**E**) The statistical results for the axon length between the two groups. Scale bars = 100 μm. (**F**) Dual two-hour intravenous anesthetics exposure had no adverse effect on neuronal migration. (**G**, **H**) Quantification of GFP+ cells in different positions. (**I**) Dual two-hour intravenous anesthetics exposure did not affect axon length of neurons in primary cultured mouse cortical neurons. (**J**) The statistical results for the axon length between the two groups. **P*<0.05, ***P*<0.01, ****P*<0.001.

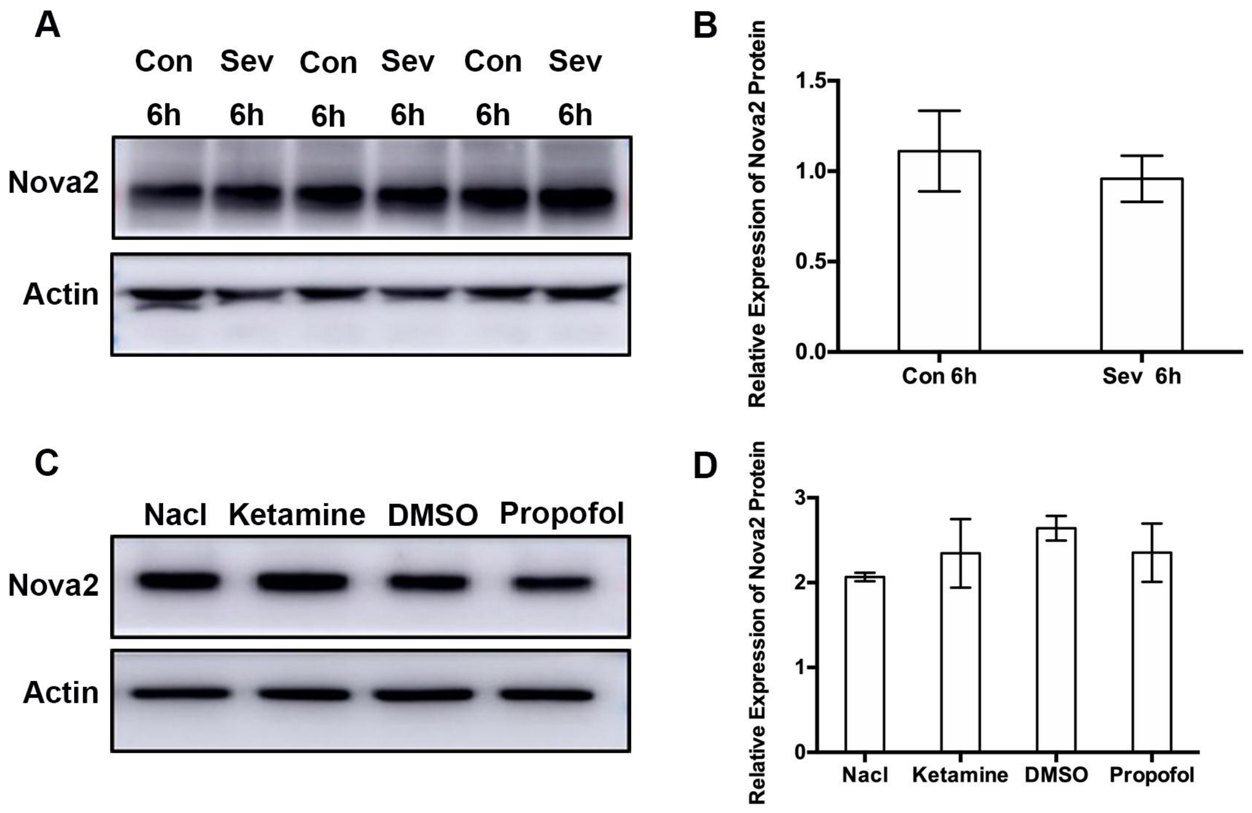

Supplementary Figure 2. Neither single long-term sevoflurane exposure nor dual intravenous anesthetics exposure affected Nova2 protein expressions in the developing mouse brain. (**A**) Western blot analysis demonstrated that single long-term sevoflurane exposure did not affect Nova2 expression in the cortical tissues of offspring mice. (**B**) Quantification of the protein expressions of Nova2 relative to Actin. (**C**) Western blot analysis demonstrated that dual intravenous anesthetics exposure also did not affect Nova2 expression in the cortical tissues of offspring mice. (**D**) Quantification of the protein expressions of Nova2 relative to Actin. **P*<0.05, ***P*<0.01, ****P*<0.001.

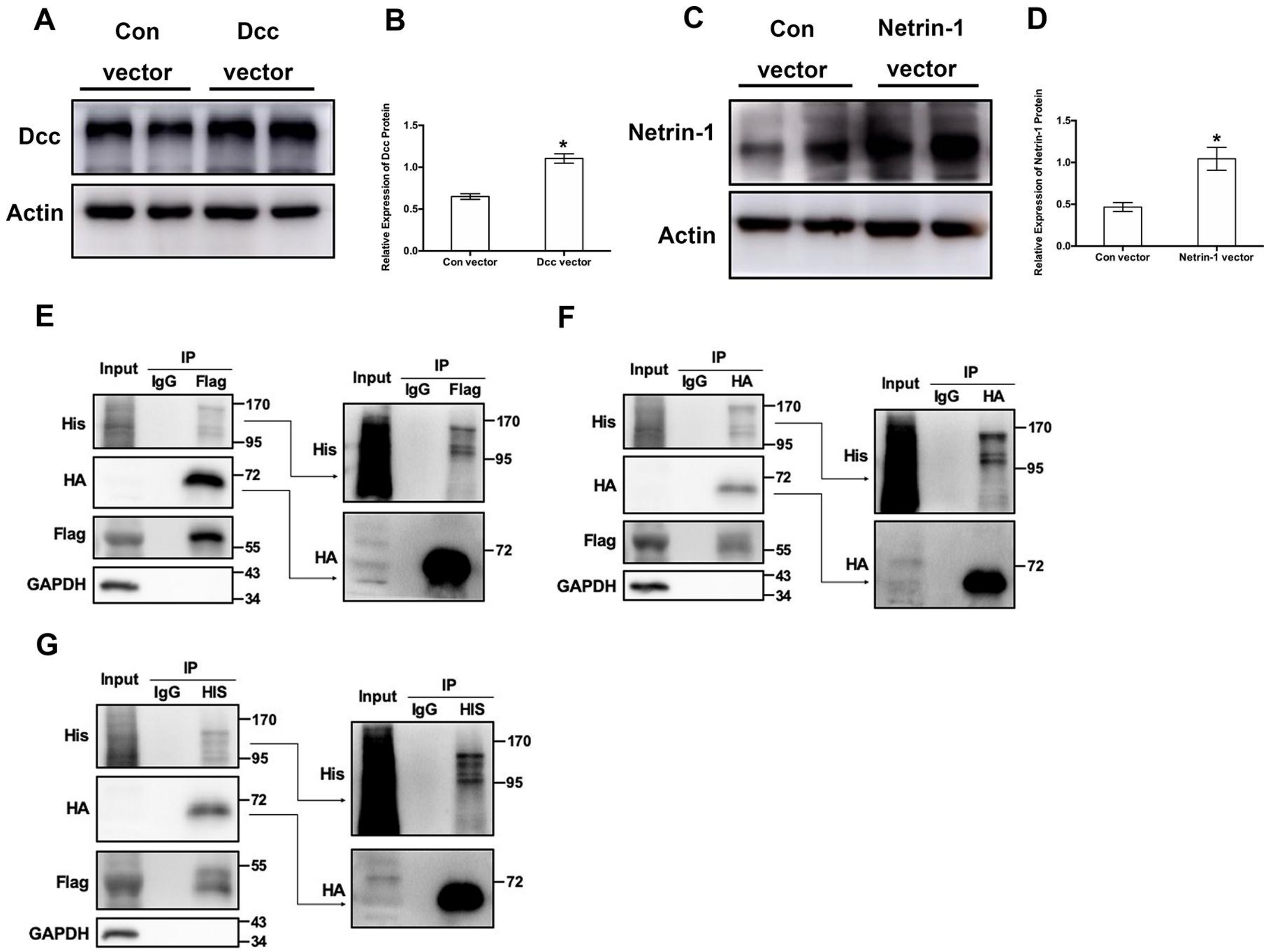

Supplementary Figure 3. Nova2 interacts with Netrin-1/Dcc in the in the cortical tissues of offspring mice. The brain tissues protein Lysate co-transfected with Flag-tagged Nova2 and HA-tagged Netrin-1/His-tagged Dcc were subjected to IP. (**A**) Dcc vector significantly increased the Dcc expression. (**B**) Quantification of the protein expressions of Dcc relative to Actin. (**C**) Netrin-1 vector significantly increased the Netrin-1 expression. (**D**) Quantification of the protein expressions of Netrin-1 relative to Actin. (**E**) IP assay used antibody against Flag. Western blotting using His and HA antibodies showed that Netrin-1/Dcc existed in the complex. (**F**) IP assay used antibody against HA. Western blotting using His and Flag antibodies showed that Nova2/Dcc existed in the complex. (**G**) IP assay used antibody against His. Western blotting using HA and Flag antibodies showed that Nova2/Netrin-1 existed in the complex. IgG was used as a nonspecific control. An input protein (5%) was used as the control in the transfected brain tissues lysates GAPDH was used as a loading control. **P*<0.05, ***P*<0.01, ****P*<0.001.
